# “I wanted information”: navigating breast Cancer and its treatment in Lima, Peru

**DOI:** 10.1186/s12905-023-02321-3

**Published:** 2023-05-04

**Authors:** Brittany C. Fields, Rachel M. Morse, Emma Ortega, Kristen Waterfield, Bryn A. Prieto, Richard Oberhelman, Valerie A. Paz-Soldán

**Affiliations:** 1 Tulane University School of Public Health & Tropical Medicine, New Orleans, LA USA; 2 Asociación Benéfica PRISMA, Lima, Peru

**Keywords:** Breast cancer, Social support, Symptom management, Health education

## Abstract

**Background:**

Breast cancer impacts millions of people worldwide, and in Peru, breast cancer is the most common cause of cancer related death among women. Breast cancer treatment is physically and emotionally burdensome and challenging for patients.

**Methods:**

In-depth interviews were conducted with 14 female breast cancer patients and survivors in Lima, Peru. The interviews explored four main themes: the women’s emotional experiences, coping mechanisms, resources available or needed, and advice for newly diagnosed breast cancer patients.

**Results:**

Respondents described a tremendous lack of informational support during and after diagnosis and treatment and requested more of this support from health professionals. Social support groups were helpful to participants; however, these forms of support were not available to all participants. Emotional and esteem support seemed amply received from family and friends, faith organizations, and fellow cancer patients. Participants experienced a range of emotions upon diagnosis and during treatment including fear, anxiety, difficulty accepting bodily changes, loneliness, and denial.

**Conclusion:**

Breast cancer and its treatment can be a long, emotional journey; more extensive forms of informational support could help patients cope with this process.

## Background

Breast cancer has the highest incidence rate of any cancer in the world and led to the death an estimated 685,000 women in 2020 [1]. Breast cancer in Peru accounted for 18.5% of all cancer cases among women, impacting nearly 7,000 women and leading to 1,824 deaths in 2020 [2]. Although much progress has been made in the detection and treatment of breast cancer over the past years, the impact of breast cancer on women’s lives remains substantial [3].

In mostly qualitative studies on the emotional journey and need for support resources for patients with breast cancer, patients most commonly report feelings of anxiety, depression, fear, and negative body image [4, 5]. Women entering treatment report anxiety about the unknowns of finances, mortality, treatment side effects, work disruption, and the enormity of decision-making [6, 7]. Women ending treatment describe feeling emotionally confused regarding a “new normal,” uncertainties about appearance, and new anxieties with symptom management [7, 8]. There is limited research in Latin America on this topic but similar emotions throughout women’s journeys are described [9, 10]. Some women in Latin America also described guilt about their diagnosis and a lack of autonomy [11].

Online communities, face-to-face support groups, familial support, and informational resources are associated positively with coping among breast cancer patients [12–14]. Health outcomes in cancer patients have been found to improve with the provision of social support [15]. However, research suggests that breast cancer patients in resource-limited areas, including Latin America, continue to have limited access to social support [10, 16]. Importantly, women with breast cancer also describe a lack of adequate knowledge about treatment, outcomes, and specific cancer types, and express the desire for more information on these topics [17, 18], including educational resources, awareness, and advocacy [19]. Moreover, these women expressed the need for information and resources related to their transition out of treatment, such as returning to work and symptoms of recurrence [8, 20].

Similar to many other countries in Latin America, Peru is facing a rise in both the incidence and mortality rates of breast cancer with an estimated 70% increase in deaths due to breast cancer between 2012 and 2030 [21]. Efforts have been made to enhance these figures by implementing initiatives such as the Strategic Program for Cancer Prevention and Control (Plan Esperanza) introduced in 2012, with the aim of reducing cancer-related morbidity and mortality, including breast cancer [22]. However, inclusion of the perspectives of the beneficiaries of these programs – breast cancer patients – has been lacking.

In one study considering the perspectives of women in the Amazonian region of Peru, Collins et al. [23] found that the majority of participants had limited knowledge of the main symptoms of breast cancer and of the accurate early detection measures. Only a small percentage recognized the need to access health services when symptomatic. There is limited information regarding the level of breast cancer knowledge in other regions of Peru. However, a review analyzing cancer control plans in Peru and other Latin American countries, identified multiple obstacles to breast cancer care, including low health literacy [22].

The aim of this study was to examine the challenges – emotional, coping strategies, and resources available or needed – that were faced by breast cancer survivors throughout diagnosis and treatment in Lima, Peru. During our in-depth interviews with 14 women, an overarching theme that emerged was the need for different types of support and health education about breast cancer before, during, and after treatment. This qualitative study provides guidance regarding the types of health education and health promotion activities that could be strengthened for this group of women.

## Methods

### Study setting and sample

This study took place in a range of middle- and low-income districts in Lima, Peru. In Peru, health care is offered in several types of facilities; 77.2% of the population receives government sponsored health care through the “Seguro Integral de Salud” (SIS) which is supposed to cover most health service costs for patients, 22.3% of the population is covered through employer based mandatory insurance also paid through a parallel government system of “EsSalud”, and the remainder have private health insurance [24]. The Ministry of Health has a health promotion unit that develops health education messages and materials nationwide; however, it is limited in funds and impact. Health education as a discipline of study does not exist in any university in Peru.

Snowball sampling was used to recruit participants older than 18 years of age who self-reported a past diagnosis of breast cancer; only women were identified. All participants were Spanish-speaking. Five interviewees were women of middle socioeconomic status, determined based on the location and condition of their residence. The remaining nine interviews were conducted in lower income neighborhoods – regions on the outskirts of Lima considered low income compared to the rest of the city, and that initiated as squatter settlements in the 70s and 80s. The age of interviewees ranged from 43 to 74 years at the time of the interview. Only one woman was still undergoing treatment at the time of the interview, whereas four interviewees had been cancer free for ≥ 5 years (“survivors”), and nine were in remission (completed treatment but not yet cleared of cancer for ≥ 5 years). Overall, stage of treatment varied from having completed chemotherapy in the month prior to the interview to having completed all treatment (surgery, chemotherapy, and/or radiation) 19 years prior to the interview. The majority (nine) detected their own lump via self-exam, whereas the remaining five were detected by a health professional.

### Data collection and analysis

Fourteen in-depth interviews were conducted using a semi-structured interview (SSI) guide in Spanish by a graduate student with previous work experience in Peru. All interviews were conducted in women’s homes. The SSI guide covered four main topics: emotional experiences upon diagnosis and through treatment, coping mechanisms, resources available or that they would have liked to have, and advice to other women with a recent diagnosis of breast cancer. Interviews were recorded using a digital recorder, and detailed notes were simultaneously taken by a research assistant hired to assist in the process. Two interviewees declined to be recorded, citing “personal reasons”. Interviews were conducted with only the interviewee, the interviewer, and the research assistant present. In one case, the interviewee’s husband was present to help her hear and understand the interview question, as she self-reported being hard-of-hearing.

Coding was both deductive and inductive. A coding guide was created based on the four main themes (deductive), and subthemes were added as they emerged inductively from the collected data; data from subsequent interviews were analyzed and new categories were added to the initial coding guide if any had been missed. Early transcripts were revisited to ensure that these were coded based on the final version of the coding guide.

Social support provides a useful structure to conceptualizing how our findings regarding patients’ needs fit together; hence, we organize the results based on four of the five types of social support: informational, social network support, emotional, and esteem (encouragement and confidence-building) support. Due to insufficient information on tangible support, this was not included in the results.

### Ethics

This study was approved by the Institutional Review Boards of both Tulane University (protocol 315711-11) and the Asociación Benéfica PRISMA (CE 0957.12), the collaborating organization in Lima, Peru. All the steps and methods were performed in accordance with the relevant guidelines and regulations. Informed written consent to participate in the study was obtained from all participants.

## Results

### Emotional experiences

The majority of women primarily reported experiencing fear at diagnosis, both for their personal fate and for that of their families, particularly their children. As one woman raising a toddler at time of diagnosis shared, *“One of the things that has made me most tense was my daughter. I thought I was going to die, and my daughter is going to grow up alone and she is a baby, no*?” (#13). Women also discussed difficulty accepting changes to their bodies resulting from removal of one or both of their breasts. Feelings of depression and loneliness generally accompanied these issues with body image, with some women reporting feeling like there was no one to understand their experience and/or that cancer was taking away too much from them. One woman stated, *“I felt mutilated”* (#8) and said that, *“To look at myself in the mirror was not easy”* (#8). Another reported feeling like *“an incomplete woman”* (#1) and even gave her spouse permission to leave her.

In addition to these common emotional experiences, women throughout the study expressed denial in accepting their diagnosis, or as one describes, *“I was silent. I did not have a reaction… I did not accept, I really did not accept the treatments, I did not accept I was sick, I did not accept anything”* (#12). Upon later being diagnosed with a recurrence, the same woman stated that she left the doctor’s office and did not return for over a year. Others mentioned that they could not believe it was happening to them; it was a surreal experience where they could not process any other information being given.

### Lack of information

All respondents reported a need for information. They reported a lack of information at each stage of their treatment: a lack of resources to confront and overcome breast cancer, a lack of guidance regarding what to expect, a lack of information as treatment ended, and even a lack of health education around prevention. Many described it as a blind process, where they arrived at each stage in treatment unsure of what to expect next, learning to cope and manage their situation along the way.

#### Misdiagnosis

Four respondents (#7, #10, #12, and #13) reported receiving an initial misdiagnosis of their cancer as *grasa* (a fat deposit) or as a benign lesion or fibroadenoma. In the search for information, another respondent called in during a health broadcast on the television and requested advice from the network’s featured physician. The physician, in turn, informed her over the phone to seek medical help based on the characteristics she described, *“Well, it is not hard, it moves… it could be a fatty cyst*” (#12). Consequently, she turned to a family physician to remove the lump, only discovering after the lump was sent for analysis by her physician that it was, in fact, cancerous.

#### Lack of knowledge among patients

Numerous respondents had never received or heard of mammograms. While every respondent had heard of cancer, the extent of their actual knowledge prior to their diagnosis varied tremendously:

*“I was one of those people who had a completely distorted perception of cancer. Initially I thought that it was only hereditary. In my family… no one had had cancer. So, I thought, ‘I’ll die of anything except cancer.’ I didn’t worry, nor did I inquire or ask anyone, nothing, and 20 years ago, there was little information, no? All I knew about cancer was that it was something serious that could kill you”* (#13).

#### Lack of information from health professionals

A frequently reported occurrence was receiving the diagnosis of cancer without any explanation from a physician or nurse regarding the implications. For those referred to an oncologist, few were aware of why they were being sent to that type of physician; one asked a friend what “oncologist” meant. Those who did not receive an explanation at the time of diagnosis reported either seeking information about their diagnosis on their own or simply accepting the diagnosis without question. Some respondents reported utilizing the Internet to find additional information about the illness. One woman stated, *“I felt that I spent lots of time informing myself… I wanted information in writing”* (#14). Some participants reached out to friends and/or acquaintances who had either a history of or more information about breast cancer. Another woman reported that she *“asked up front for more information from the doctor”* (#4). Others, however, felt they had little opportunity to ask questions of the physician, and one woman, when asked if there was any information that she would have liked to have, responded, *“I still don’t even know what I would ask”* (#8).

Another commonly reported theme was a shortage of information regarding what to expect during treatment. Frequently, the lack of information contributed to their fear and uncertainty about the illness and its treatment. One respondent stated, as advice to her physicians, *“They should be more human, tell us what we have, and not as abruptly, so that we understand, instead of leaving us with such anxiety”* (#8). Another respondent shared that having either a nurse educator or a patient advocate who provided information would be helpful. One woman (#14) was told she would need a catheter port for her chemotherapy and went out to the waiting room to ask if they could put it in – not knowing it was a surgical procedure. For most, it was not discussed upfront that each oncologist chose a different combination of chemotherapeutic drugs or requested procedures ahead of time (i.e., blood work to check for recovery) or after the fact. The lack of information continued throughout treatment for many participants, and most still had limited information related to their illness even after completion of treatment. When asked what information she would like since completing treatment, one respondent stated, *“that they had given me more information about what I have… about my diagnosis of cancer”* (#4).

The 12 women who underwent chemotherapy generally reported not knowing what to expect. One talked about being caught off guard with the side effects—mouth ulcers, diarrhea (for others, constipation), overwhelming nausea that made eating and drinking difficult. For example, she (#14) described getting mouth sores that made it painful to eat and, in mentioning this to the nurse during her next chemotherapy appointment, described being told then to “swish bicarbonate” in her mouth; the participant expressed annoyance that no one had said this to her prior to getting mouth sores. She wished she had more advice from her oncologist and nurses about what to expect and how to best manage it, before being affected by symptoms so abruptly and having to learn from other oncological patients about how they manage these problems.

For women who underwent surgery, many of them reported a lack of information about how to deal with the drains left in the surgical site immediately after: one used a small purse from a friend to store the drains at her side, another was given a special camisole with extra pockets for the storage of the drains, and another respondent shared having to utilize wider, airier clothing stating, *“And I also began, for example, to realize that I needed certain clothes, right? I had recently had my operation… I didn’t have the same mobility, I needed open clothing”* (#13). Another stated, *“I asked my doctor if I needed to bring any special clothes for after the operation. He looked at me surprised, and said ‘no’. And yet, I left with two drains hanging from me!”* (#14). Their varied knowledge about resources came from sharing experiences with other patients; none of them reported receiving this practical information regarding need for camisoles or wide clothing from their providers prior to their operations or during treatment.

#### Seeking information

For those who did seek out additional information on their treatment and its management, the resources varied from scholarly articles on breast cancer and self-help books to home remedies and information from other cancer survivors. One respondent reported finding self-help books about confronting her cancer-related emotions more helpful than information specifically about her illness. A second respondent sought information via a variety of methods: *“I still look even now, on the Internet… I read a lot of books, and I asked people that knew about breast cancer”* (#5). Two other respondents reported searching the Internet; one who spoke English found information on the American Cancer Society website, whereas the other searched with her family for herbal/plant therapies to help alleviate her treatment-related symptoms: *“Every single morning I looked* [for information] *…on fruits and vegetables… things that would help increase the hemoglobin levels”* (#2). Another respondent utilized home remedies of which she already had knowledge, stating, *“I didn’t look up additional information because I drank medicine made from tree sap, drank honey, and rubbed Copaiba tree oil on my breast”* (#1).

In many instances, participants found that useful information came from other breast cancer patients. During chemotherapy carried out in rooms with other patients, some women spoke about how this shared space resulted in information exchanges about various symptoms and how to address them: nutrition, or where to buy wigs or prosthetics, as well as obtaining emotional support from women who understood the experience. Respondents most commonly spoke about learning coping information from other patients including the benefit of using wigs, hats, and/or scarves to cover their heads, either for aesthetic reasons, practical reasons (i.e. cold weather), disguising factors (i.e., not wanting all to know they had cancer), or all of these. One respondent felt she was *“the mockery of the people here”* (#4). Another described, *“When my hair started falling out, at first I said it did not matter. When I looked at myself in the mirror, I cried and cried and went to the Central Market to buy myself a wig”* (#7). Another respondent shared, *“A scarf was more comfortable, lighter, and people would know that something was wrong, but you wouldn’t get the stares or attention that you might get from going around totally bald. It would help me get seats on combis* [minibuses], *which was very helpful since I felt so weak”* (#14). Many women also reported using makeup products to address the issues of eyebrow and eyelash loss, as well as the appearance of ashen skin.

### Social support

#### Social network support

Many respondents did not have access to formal social support systems and did not know of support groups in their health facility. These groups would have likely benefited survivors, particularly as some respondents felt as though they could not relate to those around them anymore, and others withdrew themselves to hide their illness. When asked to generally describe their experiences confronting cancer, many echoed the response that, *“It was horrible, but you have to experience it to know”* (#12). Participants also expressed frustration upon receiving advice from people who had not experienced cancer themselves: about caring for oneself, about nutrition, about alternative therapies and treatment. One respondent indicated that such advice was not appreciated and even annoying because those giving the advice had never shared her experience, nor did they have proof of such advice being of actual benefit, increasing her sense of isolation and not wanting to share more.

Participants who did have access to social support groups, spoke highly about their experiences. Three respondents described the benefits of attending regular cancer support groups; the shared experience was very therapeutic and beneficial for their own ability to overcome cancer. One respondent reported drawing strength from witnessing other patients confront their illness: *“If they could do it, then I also have to do it”* (#12). For those who perhaps did not have access to a support group and/or did not utilize one, most indicated that they would have liked to have attended such a group during treatment or even now, years after treatment. Another respondent reported having informal support group sessions with the regular faces at the chemotherapy sessions (held in a room with numerous comfortable chairs facing each other). Others found benefit in attending *charlas* (monthly group sessions) offered by volunteers at a local hospital, where topics related to cancer and its treatment were discussed. A few respondents stated that volunteering at support groups for breast cancer survivors has helped them to confront many of their difficulties post-treatment.

When asked to provide advice to future breast cancer patients, respondents demonstrated the potential benefit and emphasized the crucial role of social support in maintaining a positive outlook and staying motivated to fight. One participant stated that she was fighting for her young children; she wanted them to have a mother. On particularly bad days, she described, *“When you are down and wondering how to keep going, you sometimes have to focus on taking one step at a time”* (#14). Another stated, *“Symptoms are inevitable. Do your part”* (#8). Another respondent advised, *“Fight…. Don’t lose happiness; a smile is a great therapy”* (#7). Remaining positive while being treated with chemotherapy, radiation therapy, and surgeries can be difficult, but, as one respondent shared, *“Chemotherapy isn’t there to knock us down, but rather to cure us. Be content and confident and keep fighting to keep going”* (#7). Many participants advised trying to remain positive and confident, and despite not always feeling they were receiving the amount of information they wanted or needed, still trusting their health providers or having faith: *“Have confidence in God and in the doctor. Do everything they say”* (#6).

#### Emotional and esteem support

Most respondents reported that their greatest source of emotional support was family -- spouses, children, or other family members -- particularly with overcoming the emotional toll of dealing with breast cancer. One described the support of her daughters: *“My daughters were incredible… In that moment, they were both my mothers*” (#12). For another, the support came from her spouse: *“My husband gave me moral support. My family gave me money, visited me, called me…*” (#10). Support also came from friends in many forms. For example, one participant spoke positively about the benefit of laughter from funny videos a friend sent. When participants currently in the middle of treatment spoke about advice they would give future patients, they most commonly stated to remain positive and seek support – whether through family, friends or one’s spiritual beliefs.

Two respondents reported attending mass regularly to help them face their illness. Others reported asking family and friends to pray for them, whereas others reported praying to God, a trusted saint, or a combination of these. One respondent who reported attending mass daily, reasoned her actions by stating, *“Faith moves mountains”* (#4). One respondent reported feeling braver and that she was ready to deal with anything else because she felt that God had given her all the support she needed to survive.

## Discussion

The most striking – and actionable – finding from the interviews was the tremendous lack of informational support from health professionals. Participants reported a lack of information on a range of topics: general information on breast cancer, types of treatment they might expect and should discuss with their doctors, side effects associated with treatments and how to avoid these, preparations for surgery including what to expect right after (e.g., dealing with post-surgical tubes), emotions they might experience when treatment was over, etc. Women in this study stated that some of the more helpful information they received came from other breast cancer patients. Furthermore, a considerable proportion of women in this study were misdiagnosed. Misdiagnosis of breast cancer is also seen in number of other studies [25] including those conducted in low-income settings [26, 27]. Poor knowledge among health workers or shortages in trained personnel in low-income settings may result in misdiagnosis, though this area needs to be further explored, specifically in the context of Peru [28].

Breast cancer patients and survivors who participate in support groups report improved quality of life and less pain, which results in better compliance with medical treatment and even extended lifespan [13, 29]. Similarly, respondents in our study who participated in social support groups spoke highly about the benefit of these groups. However, we found that most of our respondents did not know about breast cancer support groups in Lima, implying that information about this type of support or its benefits is not widespread or not widely available. Organizing cancer-related support groups at oncological centers – both at public and private facilities – could be of great benefit to breast cancer patients and possibly other cancer patients, or, at minimum, having posted information about locations where these support groups are available. Several women also discussed the desire to give back to others: it could be a positive experience for all if interested breast cancer survivors are involved in a peer support role to current patients.

The programmatic implications of this finding are various. First, both public and private sector facilities can join efforts to make health education information available for women at different stages of their breast cancer journey: from diagnosis, to treatment, to what to expect as treatment comes to an end. Written health education materials that women – often in shock or denial – can refer to as the initial shock wears away or as the next step in treatment approaches could be one step to provision of information that could be useful. As mentioned, those in support groups spoke positively about these, but Lima is a large city with much traffic, and a lot of women have time or movement limitations. Offering breast cancer patients ways to connect via regular Zoom meetings or even forming chats or a Facebook page that those interested could join could also be helpful and evaluated. This would allow for the benefits of peer support, but in a way that may be more feasible for those involved. Pairing women starting the journey with women further along in the journey could also be useful for some – even if the women connect virtually versus in person.

There are several limitations associated with this study: a small sample size, our lack of use of a standardized measure of psychological distress, and our one-interview format, which did not allow us to explore certain topics more extensively, such as issues related to stress of medical bills, managing family or work lives while in treatment, managing fear of recurrence, or dealing with physical symptoms post-surgery (e.g., range of motion of arm). Another potential limitation includes respondent recall bias over the emotions they experienced, or how information availability might have changed over time, given that one participant had received treatment up to 19 years prior to the interview, which could affect recall, especially for less severe emotions that may have been experienced. An additional limitation includes the sampling strategy; in lieu of a large census to identify breast cancer patients or survivors, or identifying breast cancer patients in an oncological center, the authors used convenience and snowball sampling. Therefore, the women from each of these groups may have referred us to others with similar doctors or in the same treatment centers. This study took place in the capital city of Lima, and, with the centralization of resources and health personnel, it is most likely that the informational support needs of breast cancer patients are even higher in other urban and rural sites in Peru, outside of Lima [30]. Finally, we did not interview physicians; findings are based on women’s descriptions of their feelings and what helped.

Breast cancer and its treatment present a long and emotional journey. Expansion of informational support can facilitate a better treatment environment and promote the health and emotional well-being of breast cancer patients in Peru and similar countries in the region.

## Data Availability

Data and materials are available on request to the corresponding author.

## List of abbreviations

SIS: Seguro Integral de Salud [Comprehensive Health Insurance]
SSI: Semi-structured interviews
